# Supplementary material for: Resolving the full spectrum of human genome variation using Linked-Reads
Source: Genome Res. 2019 Apr;29(4):635–45. doi: 10.1101/gr.234443.118 (PMC6442396; doi:10.1101/gr.234443.118)
Supplement: Supplemental Material [file supp_gr.234443.118_Supplemental_longranger-2.2.zip › longranger-2.2.tar.bz2!/lr2.2-public/lib/stan/cmdstan/stan/doc/api/html/math_2matrix_2columns__dot__product_8hpp_source.html]

Stan: src/stan/math/matrix/columns\_dot\_product.hpp Source File


|  |  |
| --- | --- |
|  | Stan  2.5.0  probability, sampling & optimization |


- Main Page
- Related Pages
- Namespaces
- Classes
- Files

- File List
- File Members

All Classes Namespaces Files Functions Variables Typedefs Enumerations Enumerator Friends Macros Pages

- src
- stan
- math
- matrix

columns\_dot\_product.hpp

Go to the documentation of this file.

1 #ifndef STAN\_\_MATH\_\_MATRIX\_\_COLUMNS\_DOT\_PRODUCT\_HPP

2 #define STAN\_\_MATH\_\_MATRIX\_\_COLUMNS\_DOT\_PRODUCT\_HPP

3

4 #include <stan/math/matrix/Eigen.hpp>

5 #include <stan/math/matrix/typedefs.hpp>

6 #include <stan/math/error\_handling/matrix/check\_matching\_sizes.hpp>

7

8 namespace stan {

9  namespace math {

10

20  template<int R1,int C1,int R2, int C2>

21  inline Eigen::Matrix<double, 1, C1>

22  columns\_dot\_product(const Eigen::Matrix<double, R1, C1>& v1,

23  const Eigen::Matrix<double, R2, C2>& v2) {

24  stan::math::check\_matching\_sizes("columns\_dot\_product(%1%)",v1,"v1",

25  v2,"v2",(double\*)0);

26  Eigen::Matrix<double, 1, C1> ret(1,v1.cols());

27  for (size\_type j = 0; j < v1.cols(); ++j) {

28  ret(j) = v1.col(j).dot(v2.col(j));

29  }

30  return ret;

31  }

32

33  }

34 }

35 #endif

typedefs.hpp

stan::math::columns\_dot\_product

Eigen::Matrix< double, 1, C1 > columns\_dot\_product(const Eigen::Matrix< double, R1, C1 > &v1, const Eigen::Matrix< double, R2, C2 > &v2)

Returns the dot product of the specified vectors.

**Definition:** columns\_dot\_product.hpp:22

check\_matching\_sizes.hpp

Eigen.hpp

stan::math::size\_type

index\_type< Eigen::Matrix< double, Eigen::Dynamic, Eigen::Dynamic > >::type size\_type

Type for sizes and indexes in an Eigen matrix with double e.

**Definition:** typedefs.hpp:16

stan::math::check\_matching\_sizes

bool check\_matching\_sizes(const char \*function, const T\_y1 &y1, const char \*name1, const T\_y2 &y2, const char \*name2, T\_result \*result)

**Definition:** check\_matching\_sizes.hpp:15


---

[ Stan Home Page ]

*© 2011–2014,
Stan Development Team.*
